# Estimated Glomerular Filtration Rate Correlates Poorly with Four-Hour Creatinine Clearance in Critically Ill Patients with Acute Kidney Injury

**DOI:** 10.1155/2013/406075

**Published:** 2013-02-05

**Authors:** Christopher J. Kirwan, Barbara J. Philips, Iain A. M. MacPhee

**Affiliations:** ^1^Department of Intensive Care, The Royal London Hospital, Barts Health NHS Trust, Whitechapel E1 1BB, UK; ^2^Department of Intensive Care, St. George's Healthcare NHS Trust, Tooting, London SW17 0QT, UK; ^3^Department of Renal and Transplant Medicine, St. George's Healthcare NHS Trust, Tooting, London SW17 0QT, UK

## Abstract

*Introduction.* RIFLE and AKIN provide a standardised classification of acute kidney injury (AKI), but their categorical rather than continuous nature restricts their use to a research tool. A more accurate real-time description of renal function in AKI is needed, and some published data suggest that equations based on serum creatinine that estimate glomerular filtration rate (eGFR) can provide this. In addition, incorporating serum cystatin C concentration into estimates of GFR may improve their accuracy, but no eGFR equations are validated in critically ill patients with AKI. *Aim.* This study tests whether creatinine or cystatin-C-based eGFR equations, used in patients with CKD, offer an accurate representation of 4-hour creatinine clearance (4CrCl) in critically ill patients with AKI. *Methods.* Fifty-one critically ill patients with AKI were recruited. Thirty-seven met inclusion criteria, and the performance of eGFR equations was compared to 4CrCl. *Results.* eGFR equations were better than creatinine alone at predicting 4CrCl. Adding cystatin C to estimates did not improve the bias or add accuracy. The MDRD 7 eGFR had the best combination of correlation, bias, percentage error and accuracy. None were near acceptable standards quoted in patients with chronic kidney disease (CKD). *Conclusions.* eGFR equations are not sufficiently accurate for use in critically ill patients with AKI. Incorporating serum cystatin C does not improve estimates. eGFR should not be used to describe renal function in patients with AKI. Standards of accuracy for validating eGFR need to be set.

## 1. Introduction

There are numerous and inconsistent definitions of acute kidney injury (AKI). The RIFLE criteria [1], which were then modified to the AKIN criteria [2], form the basis for classification of AKI; however, these classifications do not provide an indication for when and how to alter the management. Their categorical rather than continuous nature is an important limitation in their use as a research tool. A more accurate real time description of true renal function in patients with AKI is needed.

In contrast, there are well-established techniques for measuring and categorizing renal function in chronic kidney disease (CKD). Glomerular filtration rate (GFR) is accepted as the best overall measure of kidney function [3, 4]. The gold standard for measurement of GFR is the urinary or plasma clearance of an ideal filtration marker, such as inulin, ^51^Cr-EDTA (^51^Cr-ethylenediaminetetra-acetic acid), DTPA (diethylene triamine penta-acetic acid), or iohexol. Measuring clearance with these markers is complex, expensive, and difficult to do in routine clinical practice [5]. As a result, clearance of the endogenous biomarker creatinine is the most widely used approach. Creatinine is found in stable plasma concentrations, freely filtered, not reabsorbed, and is minimally secreted by the renal tubule. Although not the perfect marker, it is easily measured in blood and urine and creatinine clearance (CrCl), over a defined time interval (usually 24 hours), is used as a surrogate measure of GFR. Measuring 24-hour CrCl in patients with AKI and a rapidly changing GFR will be misleading, but CrCl has been validated in various groups of patients over much shorter collection times (1, 2, 3, 4, and 8 hours) [6–10] including patients in intensive care [11], and accurate urine collection in critically ill patients is made easier by the use of urinary catheters.

Clinicians now use a set of equations [12], which have improved on the widely used Cockcroft and Galt (C&G) formula to estimate renal function in patients with CKD [13] and a GFR of 60 mL·min^−1^ per 1.73 m^2^ or less. Recently, an eGFR equation modelled to accurately predict GFR over a wider range, including values greater than 60 mL·min^−1^ per 1.73 m^2^, has been published [5], but this is not yet used in routine clinical practice. 

Some published data, albeit in abstract form, suggest that MDRD eGFR calculations may be more useful than serum creatinine alone in estimating renal function in critically ill patients when compared to CrCl measurements [14, 15]. However, these eGFR equations have not been formally validated for use in critically ill patients with AKI.

An attempt to improve the accuracy of eGFR has led to new equations for patients with CKD that incorporate the cysteine proteinase inhibitor cystatin C [16–21] (though this has had varying success). Cystatin C is constitutively expressed by all nucleated cells exhibiting a stable production rate even in the presence of an acute inflammatory response [16]. It is freely filtered by the glomerulus and almost completely reabsorbed and catabolised by proximal tubular epithelial cells [22]. eGFR equations with cystatin C as a variable have not been tested as a marker of renal function in critically ill patients with AKI.

As mentioned, CrCl measured over short-time periods (e.g., four hours) is an accepted measure of renal function in critically ill patients. It still; however, takes a significant time to process and urine creatinine measurements are often analysed once a day; thus the clinical picture may have changed by the time the result is available. There is a need for a quick and simple measurement that will provide a more precise description of renal function than AKIN/RIFLE to guide clinical practice and standardise research end points. Simple equations that have been formulated to describe renal function in CKD may have a role in describing renal function in AKI and be more sensitive than creatinine alone. 

This study aimed to test whether creatinine or cystatin-C-based eGFR equations, commonly used in patients with CKD, offer an accurate representation of 4-hour CrCl (^4^CrCl) (when less than 60 mL·min^−1^ per 1.73 m^2^) in critically ill patients with AKI. 

## 2. Methods

### 2.1. Patients and Sample Collection

This was a prospective cohort analysis of critically ill patients with AKI. Approval was obtained from a Research Ethics Committee that specialises in approving research involving patients with limited or no capacity. Written informed consent was obtained where possible. If the patient was not able to consent, written agreement for the patient to participate was obtained from a consultee in accordance with the guidelines from the Research Ethics Committee in relation to the Mental Capacity Act of 2006 (UK).

All patients admitted to the adult general ICU were considered if they had urinary and arterial catheters and fulfilled one of the AKIN criteria. Urine was collected over four hours, and a mixed sample was analysed for creatinine concentration. Serum measurements were made at the end of the collection time. Patient weight was determined from the patients themselves or their relatives or, if neither of these were possible, the most recent weight documented in the medical notes. If none of these were available then weight was estimated. Weight estimation is common in the ICU occurring in approximately 40% of admissions in our centre [23]. When required, estimation was performed jointly by the nursing and medical staff. Body surface area was calculated using the Mosteller formula [24]. Creatinine clearance was determined by multiplying the urinary creatinine concentration by the rate of urine production and dividing by the serum creatinine concentration. This was then standardised to body surface area (1.73 m^2^) for comparison with eGFR estimation.

Patients were excluded if ^4^CrCl was greater than 60 mL·min^−1^ per 1.73 m^2^ or the urine output was less than 0.24 mL·kg^−1^ per hr over the study period (i.e., <400 mL per day in a 70 kg patient's oliguria) as the clinical need to know the actual GFR is less as renal replacement therapy beckons.

### 2.2. Calculations Using Equations Which Estimate GFR

The equations used for estimating GFR are shown in Table 1 and include a comparison to serum creatinine alone [7, 14, 15, 18]. Creatinine was measured by the Jaffe reaction [25]. All these tests were performed on a Siemens ADVIA 2220 autoanalyser. Equations based on cystatin C are specific to the method used for measurement. In this study, cystatin C was measured using the particle-enhanced nephelometric immunoassay (PENIA) method and thus the eGFR equations used are those from the Levey group [16]. These appear to be more accurate than its alternative, the particle-enhanced turbidimetric immunoassay (PETIA) [26].

### 2.3. Statistical Analysis

Statistical analysis was done using Microsoft Excel and GraphPad Prism 5 (v5.03). Two-tailed *P* values < 0.05 were considered significant. Correlation coefficients (Spearman's rank) were calculated for each equation compared to ^4^CrCl. Bland-Altman analysis [27] was used to compare each equation to ^4^CrCl and the regression line plotted. Bias is the mean of the difference between the eGFR calculation and the ^4^CrCl. Percentage error or precision was calculated by dividing the 1.96 multiple of the standard deviation by the mean eGFR. Accuracy was measured as the proportion of GFR estimates within 10%, 30% and 50% deviation of the ^4^CrCl.

## 3. Results

**Figure 1:**
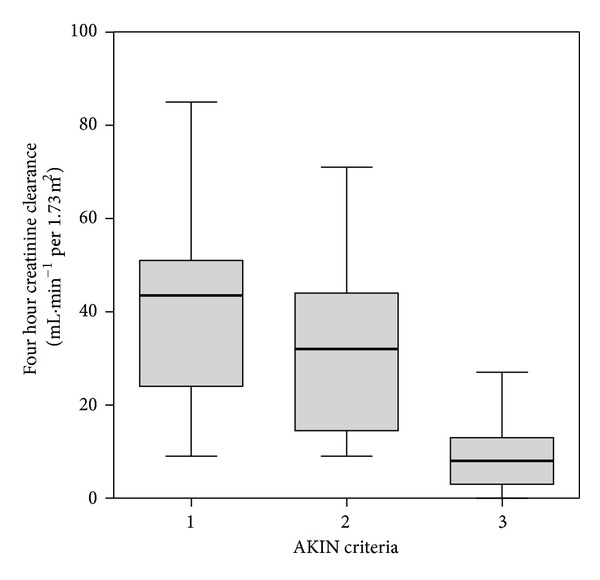
The broad range of ^4^CrCl measured across the various AKIN criteria in 51 critically ill patients with AKIN defined AKI.

**Figure 2:**
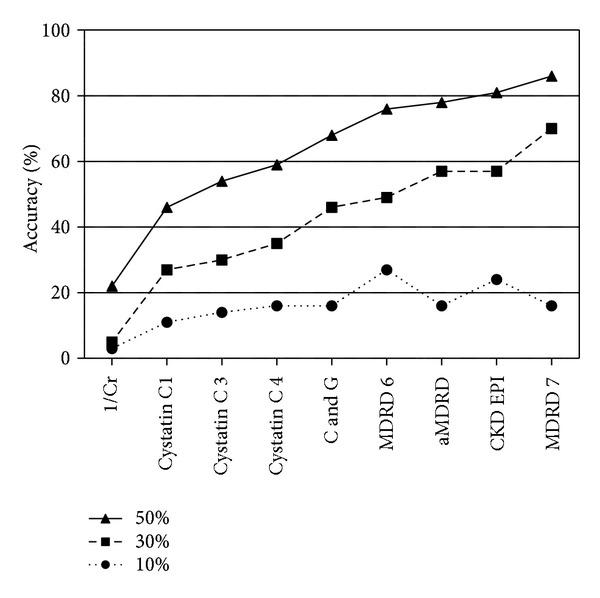
The accuracy of each of the equations expressed as the percentage of estimates within 10%, 30% (P_30_), and 50% of the ^4^CrCl.

51 patients had AKI as defined by AKIN but only 37 (20 male) of those had a ^4^CrCl of ≤60 mL·min^−1^ per 1.73 m^2^ and a urine output ≥0.24 mL·kg^−1^ per hr. There was a broad range of ^4^CrCl over each of the AKIN criteria (Figure 1). Demographics and reason for ICU admission of the 37 patients analysed are shown in Table 2. The mean (range) ^4^CrCl was 27.1 (8–51) mL·min^−1^ per 1.73 m^2^.  Table 3 summarises the performance of the eGFR equations in terms of correlation, bias, percentage error (precision), and accuracy.  Figure 2 shows the accuracy of each of the equations. The MDRD 7 equation provided the most precise and accurate estimate of creatinine clearance.

**Figure 3:**
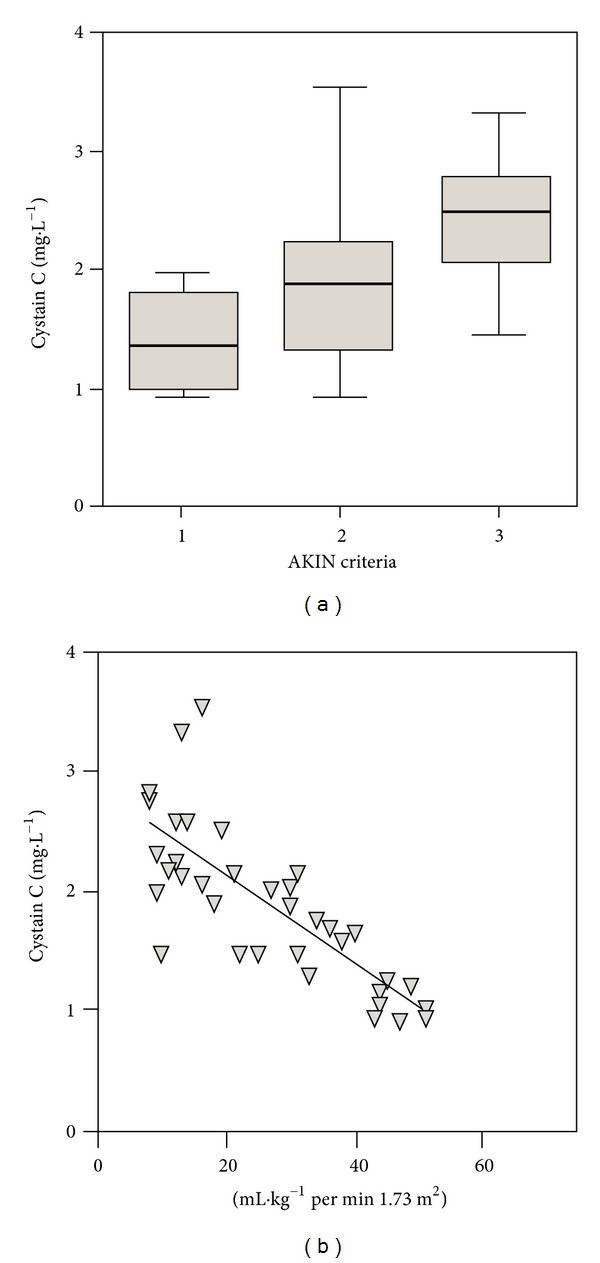
Wide range of serum cystatin C across the corresponding AKIN criteria and the correlation between ^4^CrCl and cystatin C (*r* ^2^ = 0.63; *P* < 0.0001).

Cystatin C measurements were related to the AKIN criteria and ^4^CrCl (Figure 3). There was a significant difference between 1(R) and 3(F) (*P* < 0.001). Each category had a broad range of distribution and substantial overlap between groups. Cystatin C correlated significantly with ^4^CrCl (*r* ^2^ = 0.63; *P* < 0.0001) (Figure 3). This was marginally better than creatinine alone but not as good as the correlation coefficients for the cystatin C equations. 

All the eGFR equations were better than creatinine alone at predicting the ^4^CrCl in terms of correlation, bias, precision, and accuracy. The addition of cystatin C to the calculation was better than creatinine alone but did not improve the bias or add accuracy when compared to the original MDRD equations and the new CKD-EPI equation. The MDRD 7 eGFR had the best overall combination of correlation, bias, percentage error and accuracy. 

## 4. Discussion

There is a need for a more accurate/continuous description of renal function in addition to the RIFLE/AKIN criteria for patients with AKI. For example, whereas volume overload, metabolic acidosis, hyperkalemia, and overt uraemic manifestations are commonly accepted indications for renal replacement therapy (RRT), initiation of therapy based on other conditions is more subjective and based on clinical judgement. Opinion differs on whether early RRT may benefit critically ill patients and the data up to 2007 [28] is difficult to interpret due to the large variability in the parameters used to trigger initiation of RRT [29–31]. This is supported by a more recent study involving 54 ICUs which also struggles to draw a firm conclusion about when to start RRT [32]. The ability to accurately describe renal function through the consistency of a quantitive and continuous measure of renal function may be more helpful than the descriptive scale of the AKIN criteria when constructing similar, important trials such as these.

AKI has secondary pathophysiological effects, for example, increased risk of infection [33], contribution to the development of multiorgan dysfunction syndrome [34], and altered hepatic drug metabolism [35]. Volume overload and acid-base derangements typical of renal dysfunction have serious consequences in the duration and weaning of mechanical ventilation [36]. Recent animal studies suggest that acutely ischaemic kidneys may induce both functional and transcriptional changes in the lung, independent of uraemia [37]. Metabolic disturbances and some of the other associated complications of AKI may be proportional to the severity of renal injury again reinforcing the need to explore a quantitative and continuous measure of renal function/GFR in patients with AKI.

## 5. Measuring the “Clinical Success” of eGFR Equations

This study was designed to test the potential of using readily available estimates of GFR as a bedside marker of renal function in critically ill patients with AKI to provide this measure. In analysis there is a wide variation across different measures of suitability (correlation, bias, precision, and accuracy) for these eGFR equations which in turn raises the questions; what is the best measure of suitability and what are the acceptable limits?

The correlation coefficient in all equations (including 1/creatinine) could be deemed statistically strongm but in this circumstance, it is not a good measure and is open to misinterpretation. The Bland-Altman analysis reveals wide ranges of both bias and percentage error for all equations, highlighting that a small bias does not mean a more precise equation. Acceptable limits of precision as less than or equal to 30% have been suggested in comparisons of cardiac output measures [38]. Based on these criteria, none of the eGFR equations are satisfactory. Accuracy describes at best, only 27% of all the measures of MDRD 6 within 10% of the measure ^4^CrCl with a maximum of 86% of eGFR measures by the MDRD 7 equation being within 50% of the true ^4^CrCl.

There are no defined limits of acceptability when comparing measured and estimated GFR but previous publications offer some guidance. Levey et al. [12] noted that small reductions in correlation coefficient can represent large increases in unexplained variance but continue to use this as a marker of accuracy. The paper also demonstrates that the equations perform better and are more accurate than in this study. Subsequent studies consistently publish a P_30_ (the percentage of values estimated that are accurate to within 30% of the true value) which is perhaps more useful comparison hence its use in this analysis.

A recent study of eGFR performance in renal transplant patients, [39] used Bland-Altman analysis and described the bias of C&G, aMDRD, and MDRD 7 as 15.2, 9.2, and 7.4 and worse than this study. The precision (25.4%, 21.9%, and 20%, resp.) however, was better and within a range suggested previously [38]. The percentage of values within 30% of the ^4^CrCl (P_30_) (37, 60, and 67.4, resp.) was comparable to the data from this study, and the use of the equations in renal transplant recipients is recommended.

When introduced, the CKD-EPI equation [7] had a bias of 2.1 mL·min^−1^ per 1.73 m^2^ and a P_30_ of 79.9%, which are better than data presented in this study and comparable only to the MDRD 7 equation.

Using methods based on cystatin C when compared with methods incorporating serum creatinine have shown a higher correlation and improved accuracy in predicting GFR in patients with various degrees of renal function, liver disease, and spinal cord injuries [17]. However, results in patients with diabetes, paediatric patients, and those with early renal impairment did not show a significant difference between cystatin C and creatinine based eGFR, indicating that the performance may be patient population specific [40–43]. Human studies also suggest that cystatin C can predict the development of AKI [44] and the requirement for renal replacement therapy [45], although its superiority over serum creatinine has not been a universal finding [46]. 

Data presented in this study demonstrate a very broad range of both ^4^CrCl and cystatin C measurement across each of the AKIN/RIFLE criteria. Figure 3 shows that serum cystatin C increased with worsening renal function measured by ^4^CrCl, but the correlation coefficient is not compelling and the confidence intervals are wide. When originally derived, the equations which incorporate cystatin C showed minimal bias and excellent accuracy with P_30_ of 81%, 83%, and 89% for cystatin C1, C3, and C4 equations, respectively [16]. These results were not reproduced in this study and the cystatin C equations actually perform worse than the original MDRD equations in patients with AKI.

## 6. Limitations

Measuring rapid changes in renal function accurately in critically ill patients is difficult and there is no gold standard method. A useful, routine exogenous marker has remained elusive and there are well-described difficulties when interpreting creatinine clearance. Tubular secretion and extrarenal elimination of creatinine increases as GFR deteriorates, thus exaggerating the discrepancy between the clearance of creatinine and true renal function [47]. In addition, serum creatinine concentrations are influenced by muscle mass, protein intake, gender, and age, limiting the precision further. The influence of these factors in the acute setting is not clear. However, over a period of hours and days, as the renal function deteriorates in AKI, one would anticipate that these other factors would remain relatively constant.

Aware of its limitations, in the absence of an accepted gold standard, the ^4^CrCl was piloted as a baseline standard. It incorporates both changes in creatinine and urine output and is supported by an evidence base. A small study of eighteen critically ill patients used correlation coefficients to compare clearance of DTPA or inulin (their gold-standard measure) to 2-hour creatinine clearance (^2^CrCl) [48]. The authors conclude that a ^2^CrCl is not an accurate description of inulin clearance, in this population, when the GFR is <30 mL·min^−1^. However, reanalysis of the published raw data reveals a correlation coefficient (*r*) between DTPA and 2-hour creatinine clearance of 0.92 (*P* < 0.001) though this is not discussed in the original paper. Perhaps the more encouraging conclusion should include the close relationship with DTPA clearance. There is no mention of urine volume during the study time period and patients with very low DTPA clearances (2 mL·min^−1^) were included.

On balance, until a comprehensive study is designed using a universally accepted gold-standard measure of GFR, we feel that ^4^CrCl is the most practical measure of GFR at any one time in this specific and rapidly changing clinical scenario.

This is a small single centre study but the first direct comparison of a direct measure of renal function and mathematical estimations in critically ill patients. Our conclusions are important to discourage the misinterpretation of eGFR equations in patients with AKI but also to encourage a search for a more robust method of measuring renal function other than ^4^CrCl and promote the consideration of a larger multicentre study.

## 7. Conclusion

Although our study population is small, we conclude that the MDRD equations, which estimate GFR, do not accurately predict renal function measured by ^4^CrCl and thus are not accurate enough for clinical use in critically ill patients with AKI. Although the results in this study are in fact better than any others published in critically ill patients so far, we consider that previous results have been over interpreted in favour of using GFR calculations and if a larger study was designed, a dramatic improvement in performance would be needed to show clinical relevance of these estimations. Serum cystatin C measurements, and equations which incorporate, do not add further information when estimating ^4^CrCl and should not be used in their current format in clinical practice to describe GFR.

In the future, modifications incorporating the unique circumstances of critical illness may add value to the eGFR calculations. For example, in critical illness, redistribution of albumin to the interstitial compartment causes the serum albumin concentration to be low, thus its inclusion as part of eGFR may not be helpful. Weight is also difficult to measure accurately in the ICU and patients often have many litres of excess extravascular fluid and thus even measured weight may not reflect true weight when euvolaemic. Consideration of “AKI limits” such as oliguria or anuria may also help along with, if possible, a comparison to a gold-standard measure of renal function in AKI which could confirm the validity of a ^4^CrCl. 

Finally, a consensus needs to be reached when setting performance targets of new eGFR equations. Bias, percentage error, and P_30_ are commonly reported, but a range of acceptability has not yet been set.

## Figures and Tables

**Table 1:** Equations to estimate the Glomerular Filtration Rate (eGFR) mL·min^−1^ per 1.73 m^2^.

Name	Equation		
Creatinine	$\frac{1}{\text{Creatinine}}\times 100$		
Cockcroft and Gault	$\frac{\left(140-\text{age}\right)\times \text{weight}}{72\times \text{sCr}}\times (0.85\;\;\text{if female})$		
aMDRD	175 × sCr^−1.154^ × age^−0.203^ × (0.742 if female) × (1.21 if black)		
MDRD 6	198 × sCr^−0.858^ × age^−0.167^ × sUr^−0.293^ × uUr^0.249^ × 0.822 (if female) × 1.178 (if black)		
MDRD 7	170 × sCr^−0.999^ × age^−0.176^ × sUr^−0.170^ × sAlb^0.318^ × 0.762 (if female) × 1.18 (if black)		
CKD-EPI	Female	sCr ≤ 0.7	$144\times {\left(\frac{\text{sCr}}{0.7}\right)}^{-0.329}\times 0.993^{\text{age}}\times 1.153\;\;(\text{if}\;\text{black})$
		sCr > 0.7	$144\times {\left(\frac{\text{sCr}}{0.7}\right)}^{-1.209}\times 0.993^{\text{age}}\times 1.153\;\;(\text{if}\;\text{black})$
	Male	sCr ≤ 0.9	$141\times {\left(\frac{\text{sCr}}{0.9}\right)}^{-0.411}\times 0.993^{\text{age}}\times 1.156\;\;(\text{if black})$
		sCr > 0.9	$141\times {\left(\frac{\text{sCr}}{0.9}\right)}^{-1.209}\times 0.993^{\text{age}}\times 1.156\;\;(\text{if black})$
Cystatin C 1	76.7 × Cystatin C^−1.19^		
Cystatin C 3	127.7 × Cystatin C^−1.17^ × age^−0.13^ × 0.91 (if female) × 1.06 (if black)		
Cystatin C 4	177.6 × sCr^−0.65^ × Cystatin C^−0.57^ × age^−0.2^ × 0.82 (if female) × 1.11 (if black)		

Age (years); weight (kg); sCr: serum creatinine (mg·dL^−1^) (to convert from *μ*mol·L^−1^ divide by 88.4); sUr: serum Urea (mg·dL^−1^); uUr: urine urea (g·dL^−1^); sAlb: serum albumin (mg·dL^−1^); cystatin C (mg·L^−1^); SDMA (nM·L^−1^).

**Table 2:** Patient demographics (range).

	Patients with AKI, a ^4^CrCl < 60 mL·min^−1^ per 1.73 m^2^ and a u/o > 0.24 mL·kg^−1^ per hr
Number of patients	37
Age (years)	67 (25–90)
Sex	
Male	20
Female	17
Ethnicity	
White	32
Black	3
South Asian	2
Height (cm)	170 (144–196)
Weight (kg)	82 (40–175)
Body surface area (m^2^)	1.95 (1.27–3.09)
Reason for ICU admission	
Medical	15
Elective surgery	6
Emergency surgery	16
AKIN group	
1	10
2	16
3	9

AKI: acute kidney injury; ^4^CrCl: 4-hour creatinine clearance; u/o: urine output.

**Table 3:** A summary of the performance of eGFR equations in critically ill patients with AKI, whose ^4^CrCl was less than 60 mL·min^−1^  per 1.73 m^2^ and whose urine output was greater than 0.2 mL·kg^−1^ per min during the study period (37 patients).

	^ 4^CrCl	1/creatinine	Cockcroft and Gault	aMDRD	MDRD 6	MDRD 7	CKD EPI	Cystatin C 1	Cystatin C 3	Cystatin C 4
Mean eGFR (mL·min^−1^ per 1.73 m^2^)	27.1*	53.4	35.5	33.3	35.5	28.8	32.3	43.2	41.0	39.7
Range (mL·min^−1^ per 1.73 m^2^)	8–51	13–119	11–63	9–87	9–79	8–71	9–80	17–85	16–79	15–79
*r * ^ 2^ correlation (*P* < 0.0001)		0.64	0.82	0.72	0.75	0.71	0.70	0.71	0.71	0.70
Bias		−26.3	−8.4	−6.2	−5.4	−1.6	−5.2	−16.1	−13.9	−12.5
(1.96 × SD)		28	13.72	18.6	16.66	16.6	18.33	20.14	19.36	16.6
Percentage error (precision)		52	39	56	47	58	57	46	47	42
Accuracy (%)										
10%		3	16	16	27	16	24	11	14	16
30% (P_30_)		5	46	57	49	70	57	27	30	35
50%		22	68	78	76	86	81	46	54	59

*Measured not estimated.
